# Enhancement of Dose Response and Nuclear Magnetic Resonance Image of PAGAT Polymer Gel Dosimeter by Adding Silver Nanoparticles

**DOI:** 10.1371/journal.pone.0168737

**Published:** 2017-01-06

**Authors:** Rahim Sabbaghizadeh, Roslinda Shamsudin, Najmeh Deyhimihaghighi, Arman Sedghi

**Affiliations:** 1 Physics Department, Faculty of Science and Technology, National University of Malaysia, Bangi, Selangor, Malaysia; 2 Departments of Physics, University Putra Malaysia, UPM Serdang, Selangor, Malaysia; 3 Materials Engineering Department, Imam Khomeini International University, Qazvin, Iran; Institute of Materials Science, GERMANY

## Abstract

In the present study, the normoxic polyacrylamide gelatin and tetrakis hydroxy methyl phosphoniun chloride (PAGAT) polymer gel dosimeters were synthesized with and without the presence of silver (Ag) nanoparticles. The amount of Ag nanoparticles varied from 1 to 3 ml with concentration 3.14 g/l, thus forming two types of PAGAT polymer gel dosimeters before irradiating them with 6 to 25 Gy produced by 1.25-MeV ^60^Co gamma rays. In this range, the predominant gamma ray interaction with matter is by Compton scattering effect, as the photoelectric absorption effect diminishes. MRI was employed when evaluating the polymerization of the dosimeters and the gray scale of the MRI film was determined via an optical densitometer. Subsequent analyses of optical densities revealed that the extent of polymerization increased with the increase in the absorbed dose, while the increase of penetration depth within the dosimeters has a reverse effect. Moreover, a significant increase in the optical density-dose response (11.82%) was noted for dosimeters containing 2 ml Ag nanoparticles.

## Introduction

Radiotherapy is a form of cancer treatment using high energy x-rays or gamma rays, whereby an electron beams is delivered to stop cancer cells from growing. As the absorbed radiation diminishes the cells’ ability to multiply, it can affect both cancer cells and normal tissues. The principal goal in radiotherapy is delivery of a determined dose to the tumor, while decreasing the amount of radiation that reaches normal tissues, which results in eliminating the diseased cells and improving patient’s quality of life [1]. However, before irradiating cancerous cells, the process has to be carefully planned. First, an x-ray machine and computerized tomography (CT) scanner are used to visualize the anatomical structure of the patient. These two methods allow identifying the exact position of the tumor in the body. In the course of radiotherapy treatment planning, parameters such as tumor volume distance from the radiation source to the tumor, the absorbed dose, and the characteristics of the surrounding tissues are determined. This information is input into the planning computer program, which calculates the best position of the probe to deliver high radiation doses to the tumor, while limiting the exposure of healthy tissue. For tumors that are situated deep in the body, several beams from different angles must be coordinated to obtain the best results. In order to reduce the harmful radiation effects on the patient, the dose required for eliminating cancerous cells is often delivered through several treatments. This “fractional treatment” allows time for healthy tissues to recover, while ultimately resulting in the destruction of cancerous cells. However, in order to benefit from the rapid advancement in the dynamic treatment techniques, such as stereotactic radiosurgery and intensity-modulated radiotherapy (IMRT), 3D dose distribution is required [2]. Current dosimeters such as thermoluminecent dosimeters (TLD) and radiographic film, deliver the required doses in 1D and 2D, thus limiting the potential for accurate 3D dose delivery [3]. In overcoming these limitations, polymer gel is a useful tool, as it can measure dose in 3D. The amount of produced polymer inside the gel allows a dose distribution in the 3D form, which can be visualized by MRI scan and be used in radiotherapy treatment planning.

### Polymer gel dosimeters

By knowing the energy of ionizing radiation and material composition, the absorbed dose may be calculated, which is basic information pertinent to all dosimeters. Dosimeter is a device that indicates quantifiable and reproducible changes in physical or chemical properties, which are directly related to the radiation dose delivered to the material to which it is applied. Currently utilized techniques for measuring the radiation dose can be classified into those based on absolute methods and secondary methods. While absolute methods (utilized in, for example, calorimeters, ionizing chambers, etc.) involve direct measurement of radiation dose, secondary methods, such as those implicit in radio-chromatic dosimeter film, TLD, Fricke (ferrous sulfate), polymer gel, and dosimeter, involve indirect measurements of the absolute radiation dose. Polymer gel, combined with MRI, comprises the first 3D dose distribution dosimeter.

### Nanoparticles in treatment and imaging

Applying high atomic number (Z) material as a contrast agent in low energy x-ray imaging increases the risks associated with radiology procedures. As the bone is composed of calcium, which is a high Z element, it is significantly affected by radiologic processes. Empirical evidence indicates that bone marrow absorbs high radiation doses when using x-rays in the kV range. Similarly, high absorbed dose was reported in cases where high Z materials like iodine were applied as the contrast agent. Adams, Norman, Mello and Bass [4] showed that, when ionization radiation is applied to high Z elements, it results in chromosome aberration. These authors observed that the presence of high Z material resulted in increased x-ray absorption and thus increased chromosome breakage. While these effects are undesirable in diagnostic radiology, they can be advantageous in radiotherapy. Given that high Z materials have propensity for high radiation dose absorption, loading tumors with such materials increases in the amount of dose delivered to the tumor. This strategy allows maximum dose to be received by the tumor, while reducing harm to healthy tissues.

Nanotechnology is a field that combines the latest advances in chemistry, physics, biology and medicine, with the predominant potential for early detection, accurate diagnoses, and personalized treatment of cancer [5]. Nanoparticles, especially noble nanoparticles formed by silver (Ag), gold (Au), and platinum (Pt), are versatile agents with biomedical applications in the field of sensitive diagnostic experiments, radiotherapy enhancement, and drug and gene delivery. They are proven non-toxic in gene and drug delivery applications. Characterization of nanoparticles and comparison with their bulk material counterparts revealed that they should be a preferred choice in different fields. In the field of radiotherapy, metal nanoparticles are extensively studied because of their potential application in the enhancement of the received radiation dose, with the aim of improving the treatment planning stage and determining the exact dose required for destroying tumor cells. Metal nanoparticles with high atomic number are radiation dose enhancers, whereby the metal used depends on the radiation source. Available evidence indicates that they can increase the photoelectric effect or Compton Effect, and thus increase the amount of radiation that reaches the tumors. Nanoparticles are used *in vivo* in the tumors or in dosimeters, rendering them more sensitive to the radiation dose. Radio-sensitization observed by several authors indicates that gold nanoparticles (AuNP) are the best candidates for use in radionanotherapy [6–11]. From the dosimetric point of view, the use of Au nanoparticles in radiotherapy was motivated by the fact that probability of photoelectric effect increases due to the presence of high Z (Au = 79) material inside the polymer gels, which in turn increases the absorbed dose [12]. Ag and Au nanoparticles have attracted immense attention among researchers and practitioners because of their potential application in different fields, such as chemical and biochemical sensing, biological imaging, medical diagnostics and therapeutic treatment. These two nanoparticles types have great optical properties due to excellent Surface Plasmon Resonance (SPR) [13]. Thus, in this current research, Ag nanoparticles were embedded in a PAGAT polymer gel dosimeter and were irradiated with gamma rays (γ-rays) to investigate the characteristic dosimerty of Ag nanoparticles, as these have the same specifications as Au, while being less costly.

## Experimental Procedure

### Synthesis of normoxic PAGAT gel dosimeter

In preparation for the present study, gelatin (300 bloom, Sigma Aldrich, USA) was added to the de-ionized water and left to soak for 20 min, followed by heating to 48°C. Once the gelatin was completely dissolved, the cross-linking agent, N, N’-methylen-bis-acrylamide (bis)(Alfa Aesar, Sydney) was added and stirred until dissolved. Once the bis was completely dissolved, its temperature was decreased to 37°C, after which the acrylamid (AA) (Sigma Aldrich, USA) was added and stirred until dissolved. Using pipettes, the polymerization inhibitor hydroquinone (HQ) (Fulka 98%) and the tetrakis (hydroxymethyl) phosphonium chloride (THPC)(Sigma Aldrich, USA) as anti-oxidant were combined with the polymer gel solution in a 100 ml beaker, which was sealed by aluminum foil. After preparing the solution, it was poured to 10 ml vials in dark place in order to avoid photo polymerization, and was transferred to a refrigerator, where it was stored at 10°C until needed. The optimal post-manufacture irradiation and post-irradiation imaging times, which yield the maximum ΔR_2_, were both determined to be 12 h.

### Synthesis of Ag nanoparticles

**Fig 1:**
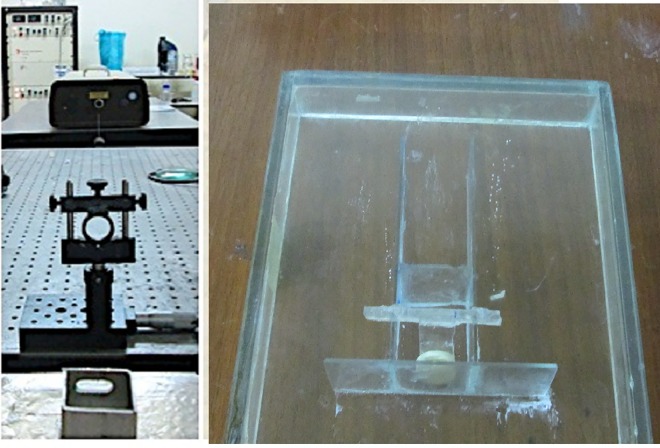
Setup of the laser and the container for fixing Ag plate.

The beam from Nd: YAG laser was focused by a lens (Focal distance (F) = 25 mm) on the surface of a silver plate (99.99%, 1.0 mm thickness) fixed inside a small container with 6 ml of distilled water. The solution was stirred by a small magnet during the interaction to ensure homogeneous distribution. The second harmonic (λ = 532 nm) was employed. The laser pulse duration was about 10 ns, the pulse-repetition rate was 10 Hz, and the maximum energy of the pulse was 0.34 J/pulse. The duration of the ablation experiment was 1 hour. It has been demonstrated that this physical method is free of any reducing agents, which are potential impurities in the nanoparticles produced, and is thus suitable for the preparation of nanoparticles of any metal element, due to the ready formation of the metal atoms by laser ablation [14]. Fig 1 shows setup of the laser and the container for fixing Ag plate. Immediately after the ablation experiment, the UV-Vis absorption spectra of Ag colloids were measured by a Shimadzu UV-1650PC spectrometer. Hitachi H-800 transmission electron microscope (TEM) was employed to take electron microphotographs of the resultant nanoparticles. For determining the exact concentration of nanoparticles inside the distilled water after laser ablation, AAS (atomic absorption spectroscopy) was utilized (Thermo scientific, S series). Atomic absorption spectroscopy (AAS) is used for detecting presence of metals in solutions. The prepared polymer gel with Ag nanoparticles was mixed completely. The nanoparticles were imbedded in the last stage of the polymer gel preparation process, after adding THPC. They were allowed to stir for at least 10 min to ensure complete dispersion. Next, the solution was poured into 10 ml vials, which were used in the assessment of different radiation doses, while the test tube of 2.5 cm diameter and 14 cm height was employed to investigate radiation penetration depth.

### Irradiation of polymer gel

The samples in the test tube were irradiated by a gamma ray source (Eldorado-8, Co-60 teletherapy unit) supplied by the Atomic Energy of Canada Limited. The dose rate of this apparatus is 0.12 Gy/min, which was calibrated by Frick dosimeter and ionization chamber. The samples were placed inside the acrylic tank, which was filled with distilled water to create a tissue equivalent when the polymer gel was irradiated. This step is required to equilibrate the secondary charge particle produced by γ-rays in the material. Irradiated and non-irradiated gel samples were scanned using a 1.5 T MRI scanner (Magneton SP Siemens, Germany). One hour before scanning small samples (10 ml vials), and two hours for bigger samples (test tube), the samples were transferred to the MRI room to equilibrate the samples at room temperature (21±0.5°C). The gels were scanned after a minimum of 12 hours.

## Result and Discussion

**Fig 2:**
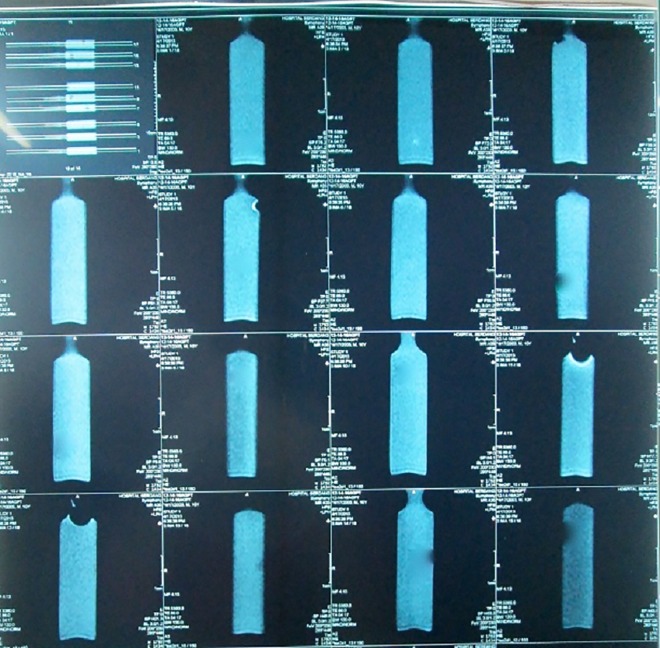
MRI images of a PAGAT polymer gel containing Ag nanoparticles.

The optical density nanoparticle scan of R_2_-weighted spin-echo for different volume of Ag nanoparticles can be seen in Fig 2 (in the form of a radiologic film). The optical density can be calculated by densitometer after printing the images on a radiologic film. The region that shows greater optical density indicates greater γ-ray absorption.

**Fig 3:**
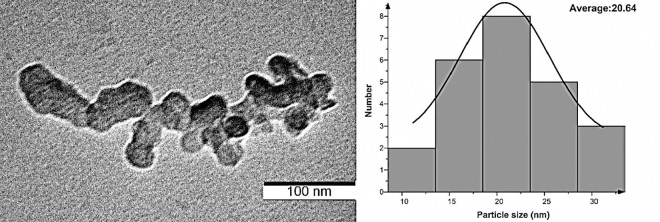
TEM image and size distribution of Ag nanoparticles.

TEM image and related particle size distribution of synthesized silver nanoparticles (Ag nanoparticles) with Nd: YAG laser are presented in Fig 3. The micrograph demonstrates that the silver nanoparticles are nearly spherical in shape with a diameter of 20 nm.

**Fig 4:**
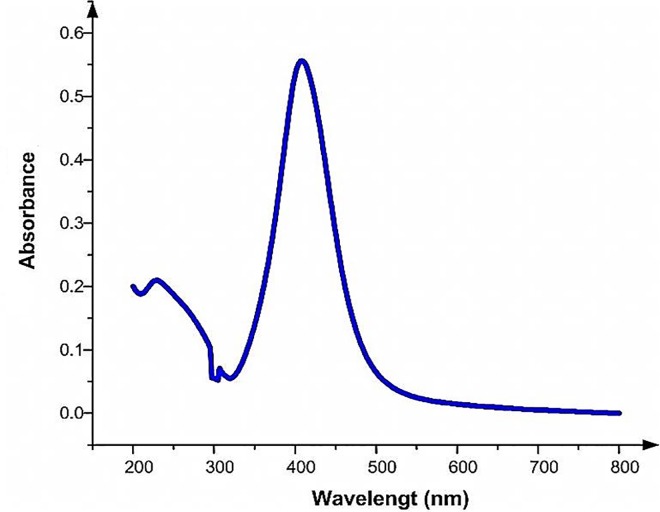
UV-Vis spectrums of Ag nanoparticles produced at 532 nm laser ablation of a silver metal plate water.

Fig 4 shows an absorption spectrum of silver nanoparticles produced at 532 nm laser ablation of a silver metal plate in water. The absorption spectrum of the solution exhibits an intense peak at 400 nm, which indicates formation of Ag nanoparticles, and a tail part of a broad band in the UV region (< 320 nm) [15].

### Optical density versus dose

**Fig 5:**
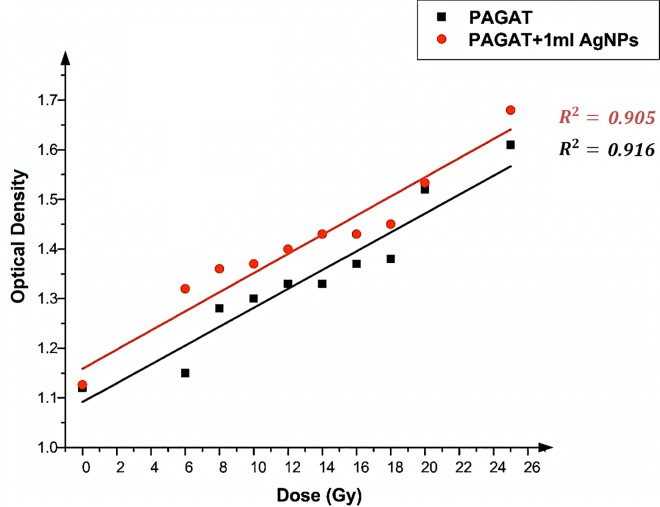
Changes in OD versus dose in the presence of 1ml Ag nanoparticles and in the absence of nanoparticles in the PAGAT polymer gel irradiated by ^60^Co.

After synthesizing the PAGAT polymer gel with optimum components and irradiating it with gamma rays, it was investigated by MRI. Many authors have investigated R_2_–dose response of PAGAT [16–18]. While the PAGAT dose response was found to be linear in the 3–30 Gy range, its response to doses below 3 Gy was not exact. Thus, in this work, to avoid non-linearity issues, we utilized doses above 5 Gy. Azadbakht and Adinhvand [19] proved that the radiation absorption of the PAGAT polymer gel was independent of the dose. Therefore, the dose was varied from 5 to 25 Gy by using a gamma cell, revealing an upward trend in the absorption with the increase in dose. This phenomenon occurs because an increase in the formation of free radicals results in an increase in the polymerization rate. As Venning [1] obtained the optimum values of all PAGAT polymer gel components; the same design was employed in this research for synthesizing the PAGAT polymer gel. The different volume of Ag nanoparticles (1, 2 and 3 ml) were embedded into 100 ml of the PAGAT polymer gel, with the 3.14 g/l as the final concentrations of Ag nanoparticles in the polymer gel. At first glance, the graphs plotting the irradiation amount vs. dose indicate an upward trend, suggesting a linear relationship between delivered dose and OD. A linear model provides a good fit to the experimental data, with good correlation. Next, we examined the effect of Ag nanoparticles on the polymerization process. When observations are made before and immediately following irradiation, no differences in the OD across samples with and without Ag nanoparticles were noted. This finding implies that Ag nanoparticles and PAGAT polymer gel cannot interact chemically with each other, thus failing to produce any enhancement in the OD. Once the dose-response slope for OD versus delivered dose was calculated (the results are plotted in Fig 5), it became evident that addition of Ag nanoparticles could increase the OD. As shown in Fig 5, 1 ml of Ag nanoparticles enhanced the OD by an average of 5.98%, with the maximum (14.78%) achieved at 6 Gy and the minimum (4.35%) at 25 Gy (Table 1).

**Table 1:** Percentage of OD enhancements after adding 1 ml of Ag nanoparticles to the PAGAT polymer gel.

Dose (Gy)	OD (PAGAT)	OD (PAGAT+1ml AgNPs)	Enhancement (%)
6	1.15	1.32	14.78
8	1.28	1.36	6.25
10	1.3	1.37	5.38
12	1.33	1.4	5.26
14	1.33	1.43	7.52
16	1.37	1.43	4.38
18	1.38	1.45	5.07
20	1.52	1.533	0.86
25	1.61	1.68	4.35
			Average: 5.98

**Fig 6:**
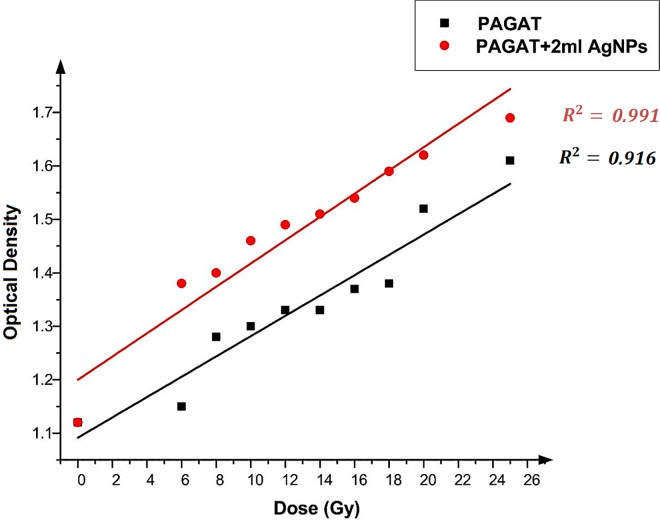
Changes in OD versus dose in the presence of 2 ml Ag nanoparticles and in the absence of nanoparticles in the PAGAT polymer gel irradiated by ^60^Co.

Fig 6 demonstrates that the same trend in enhancement was achieved when 2 ml of Ag nanoparticles were utilized. Moreover, a significant difference in response obtained from gels with and without nanoparticles can be seen. More specifically, addition of 2 ml Ag nanoparticles can improve the OD by 11.82% on average. Further details are given in Table 2, which presents the OD for each dose in the 6 to 25 Gy ranges, along with the percentage of OD enhancement, which is in the 20% to 4.97% range, respectively.

**Table 2:** Percentage of OD enhancements after adding 2 ml Ag nanoparticles to the PAGAT polymer gel.

Dose (Gy)	OD (PAGAT)	OD (PAGAT+2ml AgNPs)	Enhancement (%)
6	1.15	1.38	20.00
8	1.28	1.4	9.37
10	1.3	1.46	12.31
12	1.33	1.49	12.03
14	1.33	1.51	13.53
16	1.37	1.54	12.41
18	1.38	1.59	15.22
20	1.52	1.62	6.58
25	1.61	1.69	4.97
			Average: 11.82

**Fig 7:**
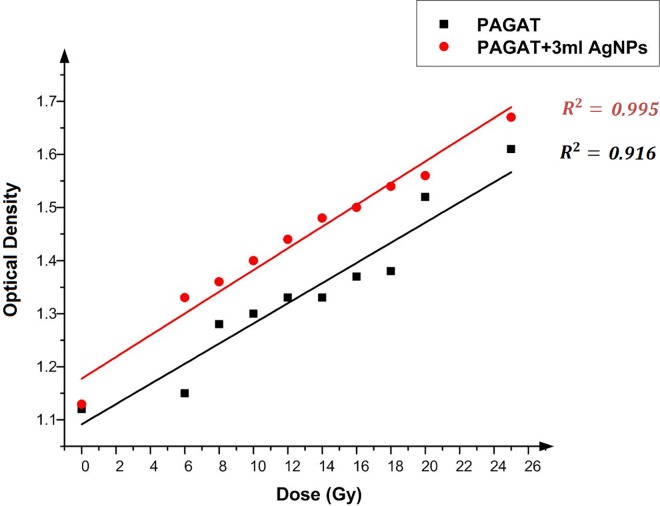
Changes in OD versus dose in the presence of 3 ml Ag nanoparticles and in the absence of nanoparticles in the PAGAT polymer gel irradiated by ^60^Co.

Fig 7 depicts the OD versus dose in the presence of 3 ml Ag nanoparticles. The upward trend due to the presence of nanoparticles is the same as that discussed above. According to Table 3, at 6 Gy, inclusion of 3 ml Ag nanoparticles can enhance the dose by 15.65%, while the OD is improved by 3.73% at 25 Gy. On average, presence of 3 ml Ag nanoparticles in the PAGAT polymer gel can enhance the OD by 8.51%.

**Table 3:** Percentage of OD enhancements after adding 3 ml Ag nanoparticles to the PAGAT polymer gel.

Dose (Gy)	OD (PAGAT)	OD (PAGAT+3 ml AgNPs)	Enhancement (%)
6	1.15	1.33	15.65
8	1.28	1.36	6.25
10	1.3	1.4	7.69
12	1.33	1.44	8.27
14	1.33	1.48	11.28
16	1.37	1.5	9.49
18	1.38	1.54	11.59
20	1.52	1.56	2.63
25	1.61	1.67	3.73
			Average: 8.51

It is obvious that Ag nanoparticles can enhance the OD, because the Compton interaction occurs between a photon and free electron (as the electron binding energy is much lower than the energy of incident photon). Therefore, the energy of incident photon must be greater than the binding energy of electron for electron displacement to occur. This is in contrast to the photoelectric effect, which is most probable when the energy of incident photon is equal or slightly greater than the binding energy of the electron. Thus, as the photon energy increases beyond the binding energy of the K-shell, the photoelectric effect decreases rapidly and the Compton effect becomes more probable, as noted in Equation 1 below.

$$\text{photoelectric}\propto Z^{3}/E^{4}$$

Where Z is atomic number and E is energy of primary photon. Therefore, the photoelectric absorption process is more likely when lower energy photons are incident on materials with high Z. In the case of γ-rays, the Compton Effect is predominant [20], as the mean energy of gamma rays emitted by ^60^Co is 1.25 MeV and the binding energy of the K-shell in the Ag atom is 25.51 KeV. The K-shell energy is too low compared to that of the incident beam. Moreover, as was explained earlier, as Compton Effect occurs in the presence of free electrons, it is independent of the atomic number (Z). This assertion is supported by the fact that Compton attenuation coefficient (σ / ρ) is independent of Z, while it depends upon the number of electrons per gram of a particular material. However, while the number of electrons per gram decreases gradually with the atomic number, most materials (except hydrogen) have approximately same number of electron per gram [21]. Thus, although (σ / ρ) is the same for all materials, the differences arise due to different number of electrons per cubic centimeter of a particular material (ρ_e_), as shown in Equation 2.

$${\text{N}}_{0}=\frac{{\text{N}}_{\text{A}}\;\text{Z}}{\mu }$$

Where *N*_0_ is number of e/g, *N*_*A*_ is Avogadro’s number, Z is atomic number and μ is molar mass. Using Eq. (2), N_0_ for Ag is calculated at 2.62 ×10^23^. If multiple density of Pt (ρ = g/cm^3^) in N_0_ (e/g), it is obtain number of electron per cubic centimeter (ρ_e_ = e/cm^3^). As the density of Ag is 10.49 g/cm^3^, then ρ_e_ for Ag will be 2.75 ×10^24^. Table 4 and Table 5 show N_0_ and ρ_e_ of various metals and body tissues, respectively.

**Table 4:** Number of electrons per gram and per cm^3^ in various metals.

Material	Density (g/cm^3^)	Z	N_0_ (e/g)	ρ_e_ (e/cm^3^)
Platinum	21.45	78	2.41 ×10^23^	5.17 ×10^24^
Gold	19.3	79	2.41 ×10^23^	4.66 ×10^24^
Silver	10.49	47	2.62 ×10^23^	2.75 ×10^24^
Lead	11.3	82	2.38 ×10^23^	2.69 ×10^24^
Copper	8.9	29	2.75 ×10^23^	2.45 ×10^24^

**Table 5:** Number of electrons per gram and per cm^3^ in body tissues.

Material	Density (g/cm^3^)	Effective Z	N_0_ (e/g)	ρ_e_ (e/cm^3^)
Fat	0.916	5.92	3.48 ×10^23^	3.18 ×10^23^
Muscle	1	7.42	3.36 ×10^23^	3.36 ×10^23^
Bon	1.85	13.8	3 ×10^23^	5.55 ×10^23^

**Fig 8:**
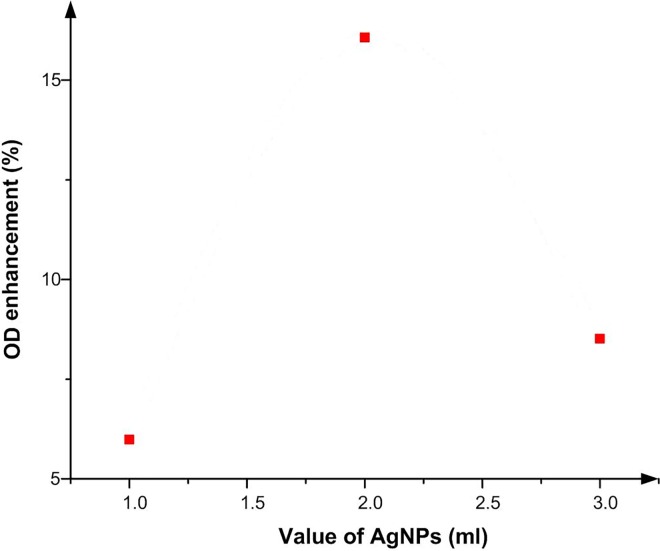
Diferrent volume of Ag nanoparticle versus percentage of OD enhacement.

**Fig 9:**
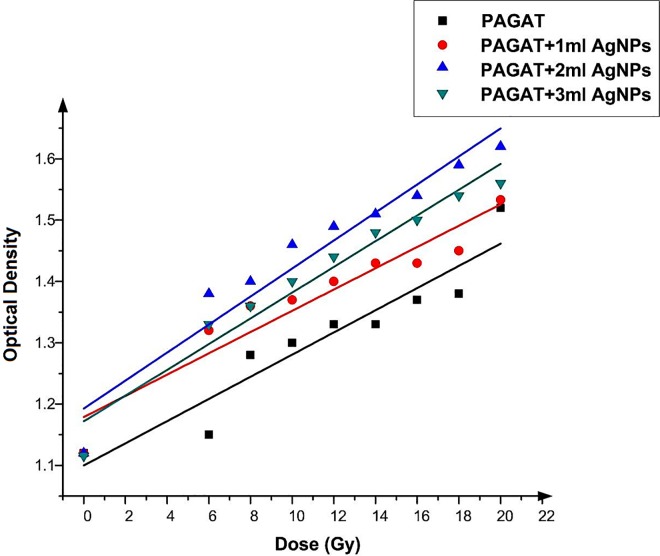
Comparison of all tested Ag nanoparticle volume.

As noted earlier, Compton Effect is the predominant effect when γ-rays are applied. The ρe for Ag is 2.75×1024 (Table 3); however, ρe in Ag is small compared to Au (4.66×1024). According to Tables 4 and 5, the difference is particularly pronounced when compared to body tissue, with fat (3.187×1023) and muscle (3.36×1023). In other words, ρe is higher in Ag than in soft tissue; thus, Ag nanoparticles can enhance the OD as a function of the radiation dose. Fig 8 provides an overview of different volume of Ag nanoparticle (1, 2 1 and 3 ml) within the polymer gel, and their effect on the OD enhancement. The peak in this graph is achieved at 2 ml of Ag nanoparticles, which is thus the optimum concentration. In addition, according to Fig 9, which is superimposes all previous graphs, using 2 ml of Ag nanoparticles in the gel results in the greatest OD enhancement, followed by 1 and 3 ml. These results are attributed to the affinity of Ag nanoparticles for free radicals (H, OH or $e_{aq}^{-}$). These free radicals are either a byproduct of radiolysis of water by ionizing radiation [22], or form an active head (carbonyl or amine) in the polymer chain, thus preventing the polymer from initiation. This, in turn, prevents polymer growth, or links it with a monomer before irradiating the sample and reducing the amount of monomers that can attend to the polymerization process. This phenomenon is more pronounced at greater Ag nanoparticle concentrations. It is more apparent when 3 ml of Ag nanoparticles is utilized that the polymerization as the dose indicator factor decreases compared to 2 ml Ag nanoparticles. However, when 2 ml of Ag nanoparticles is imbedded in the gel, 11.82% polymerization is achieved, which is well below that achieved when Au (106%) is used, as discussed before. In many applications, silver is a preferred material, such as sensing and imaging, due to its extraordinary optical properties. It possesses many advantages over Au, such as higher extinction coefficient, higher ratio of scattering to extinction and high field enhancement. On the other hand, Au has greater chemical stability compared to Ag [23]. Still, as the cost of Ag is much lower relative to Au, it is more economical to utilize it as a dose enhancer. Alternatively, the nanoparticles can be synthesized with Ag and Au plates to adjust the price and enhancement simultaneously.

### Optical density versus depth

**Fig 10:**
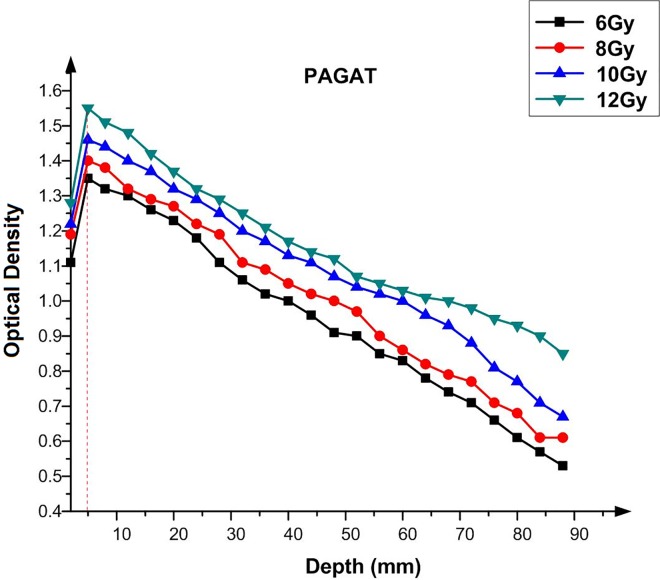
OD versus depth in the PAGAT polymer gel.

**Fig 11:**
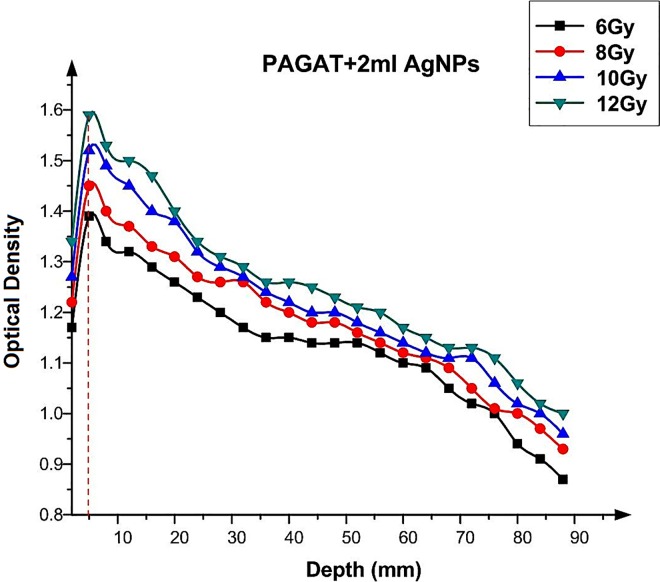
OD versus depth in the PAGAT gel with 2 ml Ag nanoparticles.

Determining the most optimal tumocidal dose is crucial in radiotherapy. As the electron and proton beam is incident on the material surface, the absorbed dose varies with the penetration depth and depends upon several factors, such as depth, energy, source to surface distance (SSD) and field size. While the measurement of in-patient dose is affected by depth, the dose distribution depends on the aforementioned factors. As it is difficult to measure the dose at the maximum depth by TLD because of its size, polymer gel as a human analogue is used in calculations [24]. Therefore, in the present study, we examined the effect of utilizing Ag nanoparticles in the PAGAT polymer gel on the dose at the maximum dose depth using tissue equivalent phantom, to determine the OD as indicated by the dose at different depths using γ-ray. The results are depicted in graphs, which show the relationship between OD and depth. It can be seen in Fig 10 that the maximum OD, as a function of dose, for PAGAT is achieved at the 0.5 cm depth for γ-rays emitted by ^60^Co [16]. In addition, while OD increases with the dose, the maximum OD is always attained at 0.5 cm depth. The only difference is that, at higher doses, the OD at 0.5 cm will be higher, as shown in Fig 11. This trend was observed when Ag nanoparticles were imbedded in the PAGAT polymer. As can be seen, addition of 2 ml Ag nanoparticles yields the best results. The maximum OD is obtained at 0.5 cm depth, the value of which varies with the radiation dose.

**Fig 12:**
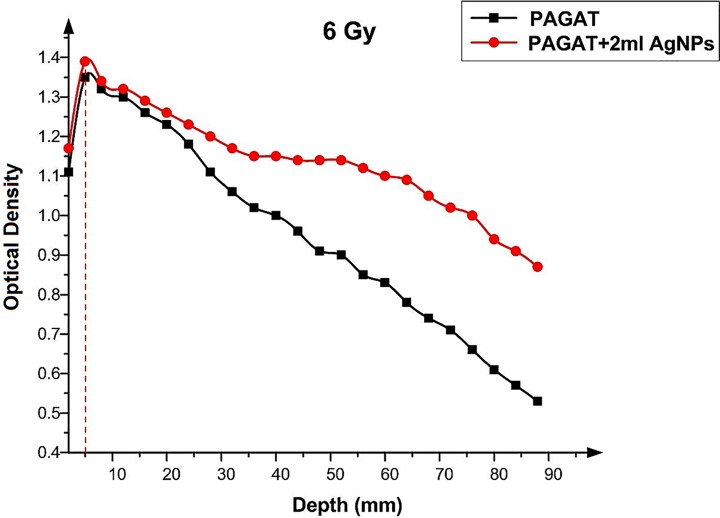
OD versus depth in the PAGAT polymer gel and PAGAT with Ag nanoparticles iraddiated by 6 Gy of ^60^Co.

**Fig 13:**
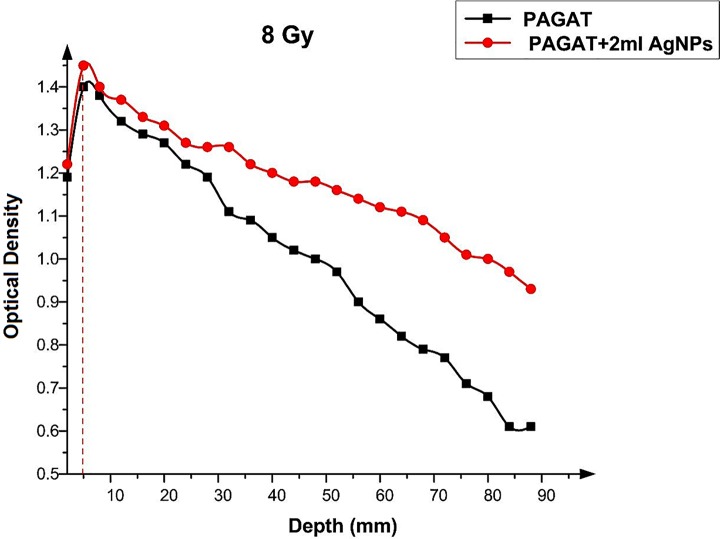
OD versus depth in the PAGAT polymer gel and PAGAT with Ag nanoparticles iraddiated by 8 Gy of ^60^Co.

**Fig 14:**
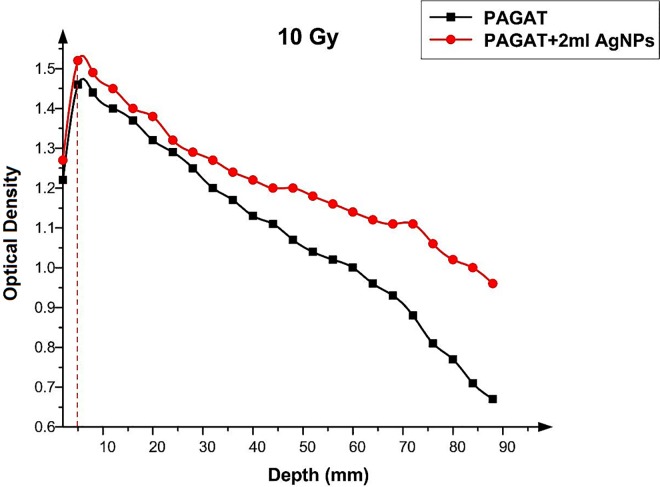
OD versus depth in the PAGAT polymer gel and PAGAT with Ag nanoparticles iraddiated by 10 Gy of ^60^Co.

**Fig 15:**
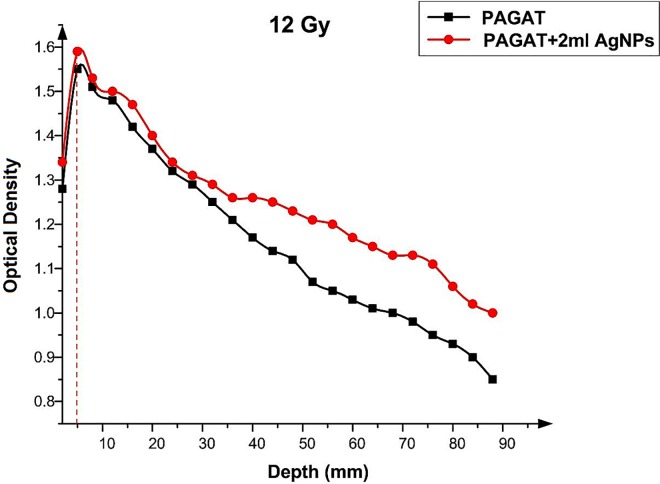
OD versus depth in the PAGAT polymer gel and PAGAT with Ag nanoparticles iraddiated by 12 Gy of ^60^Co.

Figs 12–15 compared the OD versus depth in the presence and absence of Ag nanoparticles at various doses. As can be seen, while Ag nanoparticles disperse in the PAGAT polymer gel and can enhance the OD relative to the OD obtained with the PAGAT polymer gel alone, the d_max_ remains unchanged. Table 6 provides the difference between PAGAT and PAGAT in which 62.8 mg/l of Ag nanoparticles is embedded, at the 0.5 cm depth at various doses (0 to 12 Gy). As can be seen from the tabulated data, the average enhancement at 0.5 cm that the addition of Ag nanoparticles into the PAGAT polymer gel can create is 2.64%.

**Table 6:** Comparison between OD in the PAGAT polymer gel and that obtained when 2 ml of Ag nanoparticles was imbedded in the PAGAT polymer gel at the 0.5 cm depth.

Dose (Gy)	OD (PAGAT)	OD (PAGAT+2 ml AgNPs)	Enhancement (%)
0	1.12	1.12	0.00
6	1.35	1.39	2.96
8	1.4	1.45	3.57
10	1.46	1.52	4.11
12	1.55	1.59	2.58
			Average: 2.64

## Conclusions

The significance of radiotherapy in cancer treatment and importance of MRI for diagnostics created the need for advanced dosimetric system that can quantify and verify 3D dose distribution, thus enhancing the dose received by cancerous tissue, while increasing the contrast in MRI for better diagnosis. The work presented here was based on impregnating Ag nanoparticles into a polymer gel as a radio-sensitizer to increase the MRI contrast, whereby the gray scale image was analyzed by optical density. The Ag nanoparticles were synthesized by laser ablation in distilled water. The TEM was used to determine the average particle size, which was found to be 20 nm for Ag nanoparticles that were added into synthesized PAGAT polymer gel by Venning method. Different volume of Ag nanoparticle (1–3 ml) were used for dose enhancement, whereby the PAGAT polymer gel was irradiated with 6–25 Gy ^60^Co γ-rays. Compton scattering effect was the predominant effect in the polymer gel samples, which is less dependent on Z, as it is largely influenced by the number of electrons per cm^3^ of the sample material. Since the electron density of Ag is higher in comparison with that found in human muscle, inclusion of Ag nanoparticles in the samples enhanced the polymerization of the polymer gel, thereby increasing the received dose. The dose response was enhanced by 11.82% when the amount of Ag nanoparticles was increased to the optimum concentration of 2 ml. When the amount of Ag nanoparticles is increased further, they can interact with free radicals produced by radiolysis of water or interact with active head (carbonyl or amin group) in the polymer chain, thus preventing polymerization and reducing chain growth and dose response. Measurement of the penetration depth in PAGAT was also performed in the presence and absence of Ag nanoparticles. The results indicate that, in the presence of Ag nanoparticles, the maximum dose is obtained at the 0.5 cm depth. Finally, while further addition of Ag nanoparticles increases the OD, it does not increase the maximum depth.
